# A Rare Case of Multivessel SCAD Successfully Treated with Conservative Medical Management

**DOI:** 10.1155/2020/8468730

**Published:** 2020-01-11

**Authors:** Lance Alquran, Ankita Patel, Lucy Safi, Ankitkumar Patel

**Affiliations:** ^1^Department of Internal Medicine, Hackensack Meridian Health Mountainside Medical Center, Montclair, New Jersey 07028, USA; ^2^Department of Cardiology, Hackensack University Medical Center, Hackensack, New Jersey 07601, USA

## Abstract

A female patient presented with severe, symptomatic multivessel spontaneous coronary artery dissection (SCAD) with no known medical history or risk factors. The affected vessels were left anterior descending artery (LAD), right coronary artery (RCA), and the ramus. She was treated with conservative medical management. Two months later, repeat coronary angiogram to evaluate for any residual disease was performed which showed near-complete resolution of all involved vessels.

## 1. Case History

**Figure 1:**
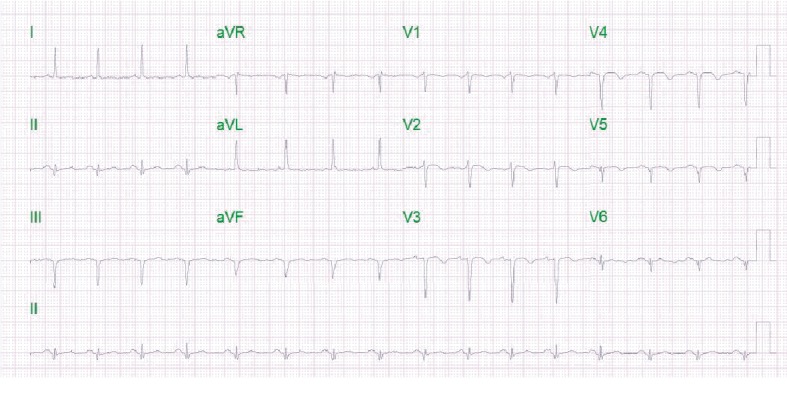
Normal sinus rhythm with an old anterior and inferolateral wall myocardial infarct.

**Figure 2:**
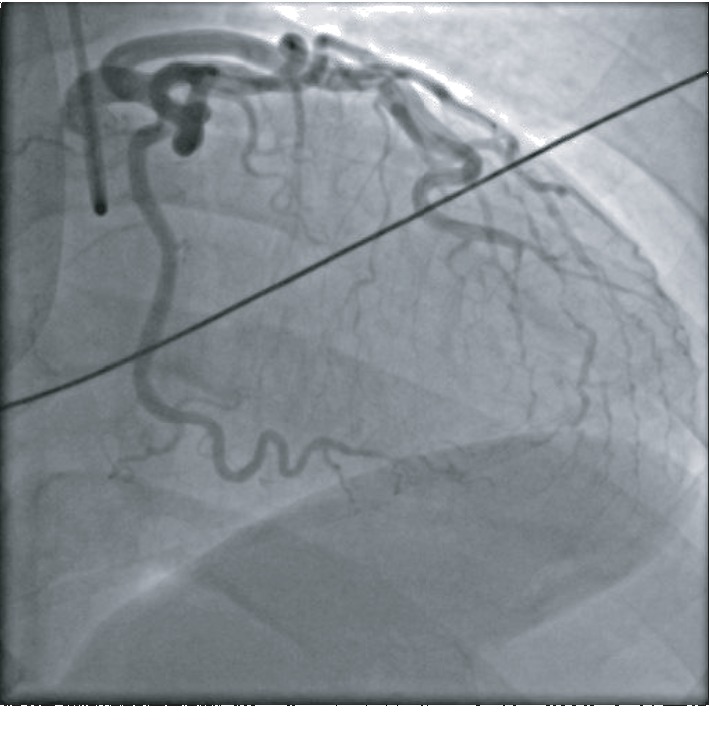
LAD with a normal caliber type 3 tortuous vessel and distal 80% long dissected section with normal apical vessel.

**Figure 3:**
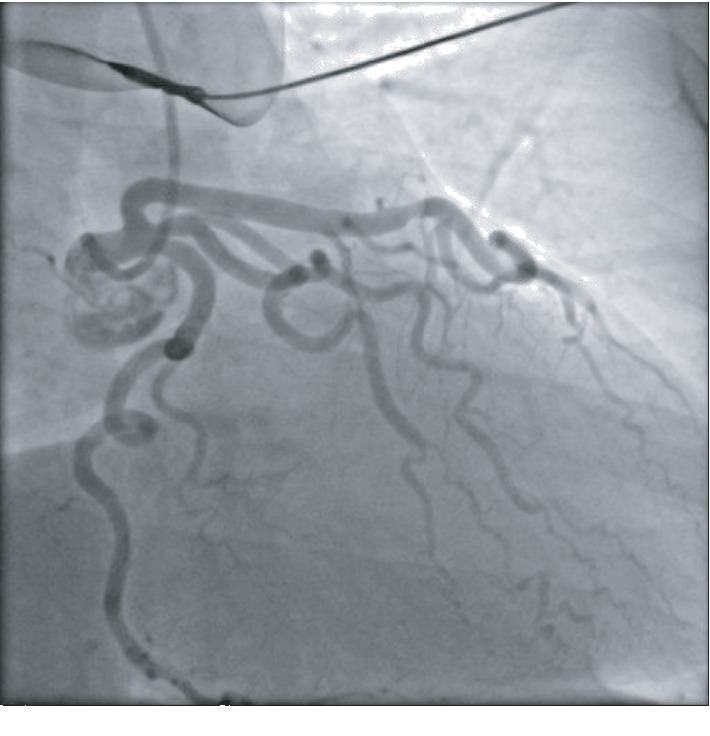
Ramus with a normal caliber tortuous vessel and distal 80% long dissected section.

**Figure 4:**
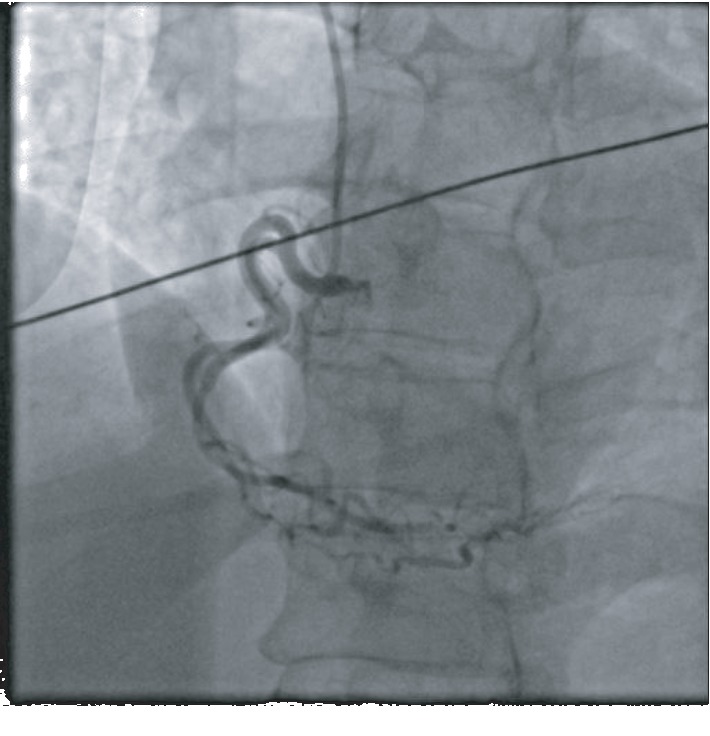
RCA with a normal caliber dominant vessel, early bifurcation, and extreme tortuosity distal area of dissection.

**Figure 5:**
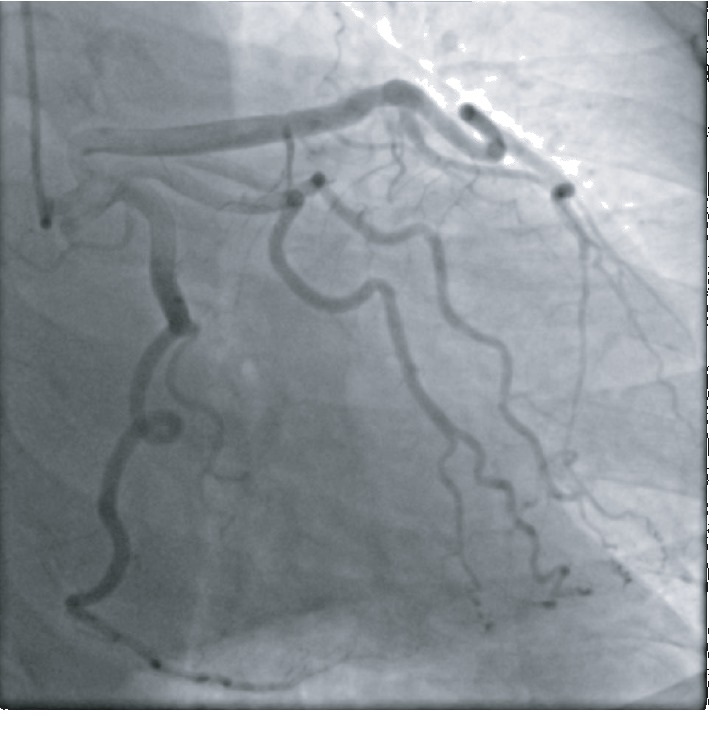
LAD with normal caliber type 3 vessel with 20% midstenosis and distal nearly completely healed SCAD long segment.

**Figure 6:**
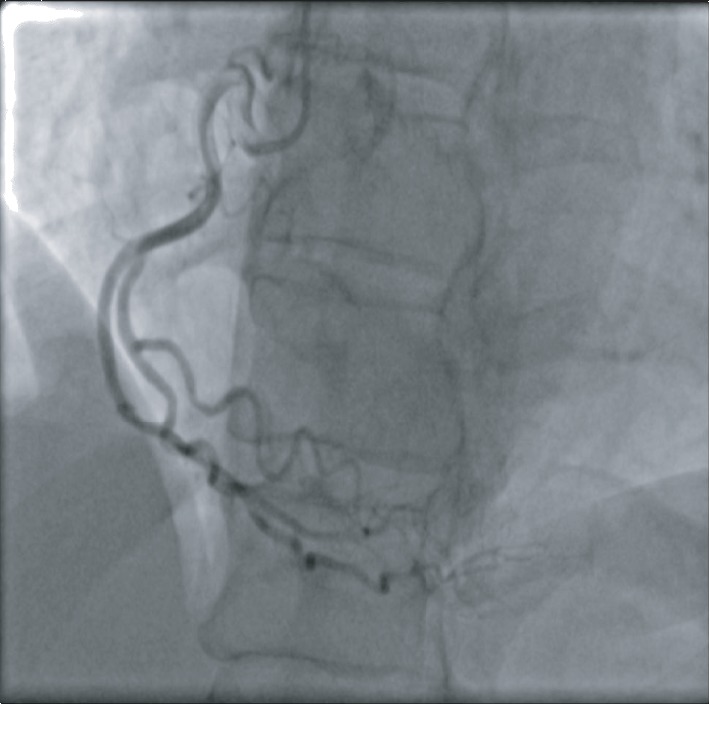
RCA with normal caliber codominant vessel with tortuosity.

**Figure 7:**
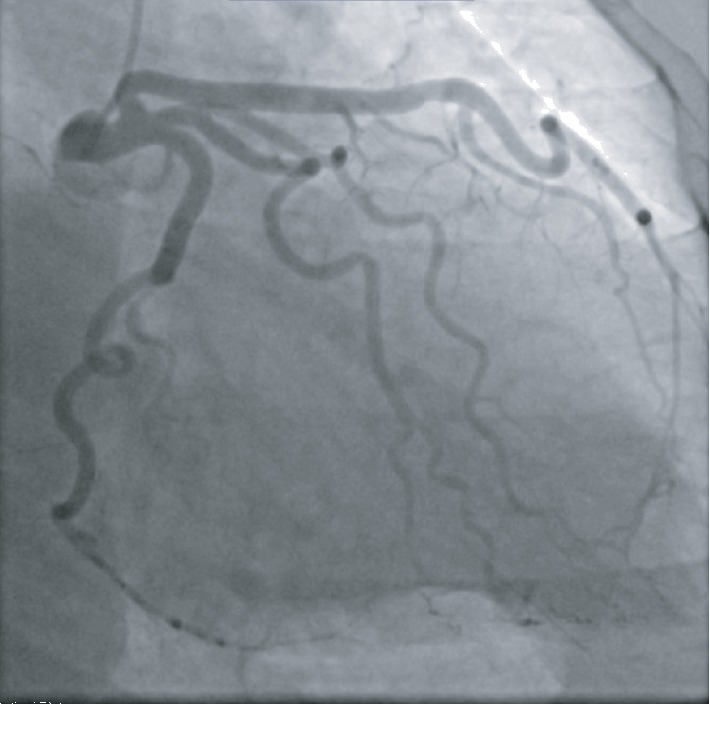
Ramus with normal caliber vessel with healed distal SCAD segments.

A 51-year-old African American female with no known past medical history presented to the emergency department with substernal chest pain, after being found in the field with frequent nonsustained ventricular tachycardia. The patient described the pain as pressure-like, moderate in intensity, which has been bothering her for the past twelve hours, alleviated by rest, and aggravated by exertion. On physical examination, she appeared anxious, well nourished, and in no acute distress. Initial vital signs showed she was hypertensive with a blood pressure of 165/94 and a regular heart rate of 89. Her neck was supple, jugular venous pressure normal, and trachea was midline. Cardiac examination revealed no abnormalities in point of maximal impulse or carotid bruits. There were no murmurs, rubs, or gallops, with normal S1/S2 components. The examination of other systems was unremarkable. In the context of substernal chest pain, the patient initially received an electrocardiogram that showed normal sinus rhythm with old anterior and inferior lateral wall myocardial infarction (Figure 1). The initial troponin upon arrival to the emergency department was 8.5 ng/mL. She was brought for an emergent coronary catheterization, for ongoing ischemic symptoms, and was found to have multivessel SCAD involving the LAD, ramus, and RCA (Figures 2–4). Echocardiogram revealed mildly depressed systolic function and regional wall motion abnormalities. Given her anatomical features, percutaneous coronary intervention (PCI) was deemed of elevated risk. Optimal medical therapy began with dual antiplatelet therapy aspirin and clopidogrel. Nitroglycerin infusion was started for symptomatic relief, as well as heparin drip for the non-ST-segment myocardial infarction. The patient developed symptom relief. Her hospital course was uncomplicated and with conservative therapy she has done well. After receiving conservative medical management, repeat coronary angiogram was preformed to access for residual disease, to see if the dissections have resolved. The repeat angiogram showed near-complete resolution of the LAD, RCA, and ramus (Figures 5–7). Repeat electrocardiogram two weeks after PCI showed no acute ST-T changes. Her New York Heart Association (NYHA) functional class was class I; she had no signs or symptoms of heart failure post-procedure. Taking into account the patient's functional status with only minor deficits on initial echocardiogram, there was no repeat scan preformed. At post-procedure, she did not have exertional angina, making her class 0 on the Canadian Cardiovascular Society grading of angina pectoris.

## 2. Discussion

SCAD is a relatively rare cause of myocardial infarction, being diagnosed in approximately 0.07-0.2% of all angiograms [1]. It is thought that the true incidence remains unclear because of the cases which go underdiagnosed [2, 3]. There has been an increased rate of diagnosis in recent years, likely secondary to increased use of coronary angiograms, and application of high-resolution intracoronary imaging [4]. Multivessel SCAD is much more rare and occurs in 9-23% of described cases [5]. Patients who are correctly diagnosed usually present with acute coronary syndrome positive biomarkers [2]. Ventricular arrhythmias have been associated with 2.8-10% cases [2, 6–8]. Patients usually present with the same signs and symptoms of acute coronary syndrome, with chest pain being the most common complaint [5]. Coronary angiogram is the first-line method used to diagnose SCAD if suspected [5]. There have been no dedicated studies regarding the role of coronary computed tomography angiography (CCTA) in the setting of acute SCAD [5]. CCTA is generally contradicted in patients with a high likelihood of acute coronary syndrome and is not recommended as a first-line investigation [5]. Also, normal findings on CCTA cannot exclude SCAD [5]. Traditionally, SCAD is described as multiple radiolucent lumens with extraluminal contrast staining that can show intraluminal filling defects or spiral dissections [9]. When treating SCAD, the goals of care are to provide symptomatic relief, prevent reoccurrence, and improve short/long-term outcomes [5]. Some experts recommend dual-antiplatelet therapy for a minimum of 1 year after diagnosis of SCAD, as well as lifetime aspirin therapy [10]. There is no clear evidence supporting the use of dual-antiplatelet therapy in patients with SCAD who did not receive coronary intervention [5]. The theoretical benefit of early dual-antiplatelet therapy is protection from subsequent thrombus formation [5]. No studies have been performed to compare the short- and long-term outcomes, as well as risk of bleeding, in patients treated with dual-antiplatelet therapy and aspirin alone [5]. Studies have shown PCI therapy to cause high rates of technical failure and does not protect against reoccurrence or vessel revascularization [6]. Conservative management with close observation can be preferred [6]. There have been no comprehensive prospective studies that performed angiographic restudy after the diagnosis of SCAD [5]. However, there is observational data that indicates angiographic healing of SCAD in (70-97%) of patients who were restudied weeks to months after being conservatively managed [5].

## 3. Conclusion

Multivessel SCAD is a rare but serious cause of ACS that requires prompt diagnosis and treatment. We describe a case of multivessel SCAD, which was treated conservatively. The repeat coronary angiogram to evaluate for any residual disease was performed two months after initial diagnosis and showed near-complete resolution of all involved vessels.
